# Clinical and Biochemical Associations with Diabetic Retinopathy in Male Patients in the Gaza Strip

**DOI:** 10.3389/fendo.2017.00302

**Published:** 2017-11-10

**Authors:** Ayman M. AbuMustafa

**Affiliations:** ^1^Department of Health Research, Human Resources Development, Ministry of Health, Gaza, Palestine

**Keywords:** diabetic retinopathy, biochemical, clinical, male, Gaza Strip

## Abstract

**Background:**

There are limited data on the prevalence and risk factors for diabetic retinopathy (DR) in the Gaza Strip.

**Objective:**

To assesses clinical and biochemical associated with DR in males with type 2 diabetes mellitus (T2DM) in the Gaza Strip.

**Methods:**

One hundred and fifty males with T2DM from the Gaza Strip underwent a questionnaire interview, serum biochemical analysis, and assessment of their previous urine and blood results.

**Results:**

The prevalence of DR was 24.7%. The duration of diabetes and prevalence of neuropathy, nephropathy, cardiovascular disease, and recurrent infections were significantly higher among patients with DR compared with those without DR (*p* < 0.05). Serum urea, creatinine, glucose, cholesterol, and low-density lipoprotein cholesterol were significantly elevated, whilst eGFR and high-density lipoprotein cholesterol were significantly lower in patients with DR compared with patients without DR (*p* < 0.05). Urinary albumin concentration and albumin creatinine ratio (ACR) was higher in patients with DR. ACR correlated significantly with the duration of T2DM (*r* = 0.311, *p* < 0.001), glucose (*r* = 0.479, *p* < 0.001), urea (*r* = 0.337, *p* < 0.001), creatinine (*r* = 0.275, *p* = 0.001), and GFR (*r* = −0.275, *p* < 0.001).

**Conclusion:**

These data show a high prevalence of DR in an unselected cohort of patients with T2DM and relationships to modifiable risk factors in Gaza.

## Introduction

The main form of diabetes mellitus is type 2 diabetes (T2DM) and affects more than 90% of cases (1). Type 2 diabetes is characterized by insulin resistance and eventually inadequate insulin production, leading to hyperglycemia (2). Chronic hyperglycemia is associated with long-term microvascular and macrovascular complications (3). Retinopathy, nephropathy, and neuropathy are common microvascular complications of T2DM (4, 5). Optimal control of blood pressure with an ACE inhibitor for microalbuminuria and laser photocoagulation for retinopathy can delay end stage renal failure and blindness, respectively (6). Retinopathy is characterized by damage to the blood vessels and neurones in the retina (7) and has been shown to be related to a longer duration of diabetes and hyperglycemia (8). Initially, there may be minimal alterations in vision, but as the condition progresses color vision may become impaired and eventually there may be total loss of vision (9). Whilst diabetic retinopathy (DR) is the most common cause of vision loss in people with diabetes mellitus; other causes, including glaucoma, age-related macular degeneration, and cataract must be assessed for (10).

Diabetic retinopathy can be diagnosed during a dilated retinal exam by an ophthalmologist or optometrist (11), but additional assessment can be undertaken to identify other pathology affecting vision (12, 13). The prevalence of DR varies in different countries and likely reflects different populations and control of risk factors for DR (14, 15). Early diagnosis and optimal control of risk factors can limit progression of DR (16). There are limited data on DR in the Gaza Strip with a previous study showing a relatively high prevalence of visual impairment and blindness and diabetes (17). A previous study has shown a high prevalence of DR affecting 27% of patients with T2DM in the Gaza Strip (18). Further data on the disease are lacking and restricted to annual reports emerging from the Palestinian Ministry of Health. Patients with T2DM in Gaza hospitals and clinics undergo rudimentary assessment of hyperglycemia with no formal assessment of complications, particularly DR. This study identifies the prevalence and modifiable risk factors for DR in the Gaza Strip.

## Materials and Methods

### Study Design and Study Population

The present study was a cross sectional study of 150 T2DM males (40–60 years old) based on current World Health Organization diagnostic criteria for diabetes (19). The patients were randomly selected from each of the five diabetes clinics in all the Governorates of the Gaza Strip: North 30 (20.0%), Gaza 50 (33.3%), Mid-zone 22 (14.7%), Khan Yunis 30 (20.0%), and Rafah 18 (12.0%). Patients with a urinary tract infection were excluded.

### Questionnaire Interview

Each patient was interviewed with a questionnaire on T2DM from the Palestinian Ministry of Health with some modifications (20). Most questions were the no/yes type, which offer a dichotomous choice (21). A questionnaire was piloted with 10 patients not included in the population sample, and modified as necessary. The questionnaire included questions related to diet, family history of T2DM, age, and smoking.

### Patients’ Records

Clinical data including duration of diabetes and diagnosed diabetic complications were obtained from the patients’ records. The body weight and height of each individual dressed in light clothing without shoes were measured using a carefully calibrated balance (Detecto, CAP-180 Kg, USA) for weight and vertical measuring rod for height and the BMI was calculated as Kilogram (kg) body mass/height in meter squared (22).

### Urine and Blood Sampling and Processing

Fasting blood samples (about 8 ml each) and random urine samples were collected and centrifuged at 4,000 rpm/10 min using a Rotina 46 Hettich Centrifuge, Japan.

### Biochemical Analysis

The glucose oxidase/glucose peroxidase (POD) method was used to measure serum glucose (Labkit Kits, Spain) (23). Creatinine and urea were determined by the urease glutamate dehydrogenase/UV method and by the Alkaline Picrate method, respectively, using the BioSystems kit, Spain (24, 25). Serum cholesterol and triglycerides were measured by the cholesterol oxidase/POD method and by the glycerol phosphate oxidase/POD method, respectively, using the BioSystems kit, Spain (26, 27). High-density lipoprotein cholesterol (HDL-C) was determined by the precipitating method using Labkit kit, Spain (28). Low-density lipoprotein cholesterol (LDL-C) was calculated using the empirical relationship of Friedewald (29).

### Urine Analysis

Urinary albumin was determined by Immunoturbidometry–Latex method using BioSystems kit, Spain (30). Urinary creatinine was measured by kinetic test without deproteinization using DiaSys reagent kits (31). Albumin creatinine ratio (ACR) (mg/g) = microalbumin in urine (mg/l) × 1,000/creatinine in urine (mg/dl) × 10. eGFR was calculated by the Schwartz equation: eGFR (ml/min/1.73 m^2^) = 0.55 × length/serum creatinine.

### Data Analysis

Data entry and statistical analyses were performed using Statistical Package for Social Sciences Inc., Chicago, IL (SPSS) computer program version 23 for windows. A simple distribution of the study variables and cross tabulation was applied. χ^2^ was used to identify the difference between variables. Means were compared by independent-samples *t*-test. Pearson’s correlation test was applied. Logistic and multiple linear regressions by backward stepwise method were also applied to build a model to predict DR. The results were accepted as statistically significant when *p* < 0.05. Range as minimum and maximum values was used. The percentage difference was calculated according to the formula: percentage difference equals the absolute value of the change in value, divided by the average of the two numbers, all multiplied by 100. Percent difference = (|(V1 − V2)|/((V1 + V2)/2)) × 100.

### Ethical Consideration

The research was undertaken according to the Declaration of Helsinki and after the Local Research Ethics Committee had approved the study. All T2DM patients provided written informed consent prior to the study.

## Results

### Clinical and Demographic

The mean age of diabetic patients was 50.6 ± 6.2 years, the mean duration of diabetes was 7.0 ± 5.8 years, and the mean BMI was 30.3 ± 7.8. 50.7% of patients with diabetes had a diabetes duration ≤5 years, 28.0% had diabetes from 6 to 10 years, the remaining 21.3% had diabetes for more than 10 years. Retinopathy occurred in 37 (24.7%), nephropathy in 71 (47.3%), cardiovascular diseases (CVDs) in 15 (10.0%), and neuropathy in 5 (10.0%) patients. Thirty-two (21.3%) of patients were smokers, 90 (60%) had a family history of diabetes, and 65 (43.3%) were on a diet.

### Clinical and Demographic Characteristics of Patients with and without DR

There were no significant differences between patients with and without retinopathy for age, BMI, and diet (*p* > 0.05) (Table 1). Significantly more patients with DR [14 (37.8%)] compared with those without retinopathy [18 (15.9%)] (χ^2^ = 7.972 and *p* = 0.005) were smokers. More patients with DR [28 (75.7%)] had a family history of diabetes compared with patients without retinopathy [62 (54.9%)] (χ^2^ = 5.029 and *p* = 0.025).

**Table 1:** Clinical and demographic characteristics of the study population.

Characteristic	T2DM		Test	*p*-Value
	Retinopathy (*n* = 37)	No retinopathy (*n* = 113)		
Age (years) (min–max)	51.7 ± 6.4 (40–60)	50.2 ± 6.1 (40–60)	*t* = −1.290	0.199
BMI (kg/m^2^) (min–max)	28.6 ± 3.4 (22.1–39.2)	30.9 ± 8.7 (19.5–108.7)	*t* = 1.518	0.131
Smoking			χ^2^ = 7.972	0.005
Yes	14 (37.8)	18 (15.9)		
No	23 (62.2)	95 (84.1)		
Family history			χ^2^ = 5.029	0.025
Yes	28 (75.7)	62 (54.9)		
No	9 (24.3)	51 (45.1)		
Diet			χ^2^ = 0.000	0.99
Yes	16 (43.2)	49 (43.4)		
No	21 (56.8)	64 (56.6)		

*People with BMI = 18.5–24.9 were considered to have normal weight, people with BMI = 25.0–29.9 were classified overweight and people with BMI ≥ 30.0 were considered obese (20). Values are *n* (%) except age and BMI where values are expressed as mean ± SD*.

*BMI, body mass index*.

### Duration of Diabetes and Diabetic Complications in Patients with and without DR

The mean duration of diabetes in patients with DR was significantly longer than those without DR (*p* < 0.001) (Table 2). The percentage of patients with CVD (*p* = 0.007), neuropathy (*p* = 0.007), nephropathy (*p* = 0.037), and recurrent infections (*p* = 0.034) was significantly higher in patients with DR compared with patients without DR.

**Table 2:** Duration of diabetes and diabetic complications in diabetic patients with and without retinopathy.

Characteristic	T2DM		Test	*p*-Value
	Retinopathy (*n* = 37)	No retinopathy (*n* = 113)		
Diabetes duration (years) (min– max)	10.5 ± 6.8 (1–23)	5.8 ± 4.9 (1–25)	*t* = −4.499	<0.001
CVD			χ^2^ = 7.371	0.007
Yes	8 (21.6)	7 (6.2)		
No	29 (78.4)	106 (93.8)		
Neuropathy			χ^2^ = 7.371	0.007
Yes	8 (21.6)	7 (6.2)		
No	29 (78.4)	106 (93.8)		
Nephropathy			χ^2^ = 4.332	0.037
Yes	23 (62.2)	48 (42.5)		
No	14 (37.8)	65 (57.5)		
Recurrent infections			χ^2^ = 4.505	0.034
Yes	6 (16.2)	6 (5.3)		
No	31 (83.8)	107 (94.7)		

*Values are *n* (%) except duration of diabetes where values are expressed as mean ± SD*.

### Metabolic Profile of Patients with and without DR

Fasting serum glucose (*p* = 0.006), urea (*p* < 0.001), creatinine (*p* = 0.006), cholesterol (*p* = 0.002), and LDL-C (*p* < 0.001) were significantly higher and HDL-C (*p* = 0.004) was significantly lower with no difference in triglycerides (*p* = 0.111) between patients with DR compared with patients without DR (Table 3).

**Table 3:** Serum glucose, urea, creatinine, and lipid profile of diabetic patients.

Variable	T2DM		% difference	*t*-Test	*p*-Value
	Retinopathy (*n* = 37)	No retinopathy (*n* = 113)			
Glucose (mg/dl) (min–max)	212 ± 88.1 (98–400)	168.7 ± 78.8 (72–460)	22.7	−2.817	0.006
Urea (mg/dl) (min–max)	28.9 ± 5.0 (23–43)	22.9 ± 8.6 (8–59)	23.2	−4.054	<0.001
Creatinine (mg/dl) (min–max)	0.7 ± 0.1 (0.5–1.1)	0.6 ± 0.2 (0.2–1.4)	15.4	−2.805	0.006
Cholesterol (mg/dl) (min–max)	214.9 ± 30.1 (175–295)	192.9 ± 39.8 (88–315)	10.8	−3.092	0.002
Triglycerides (mg/dl) (min–max)	234.5 ± 103.4 (98–759)	209.4 ± 73.1 (66–371)	11.3	−1. 602	0.111
HDL-C (mg/dl) (min–max)	40.4 ± 4.1 (30–49)	43.7 ± 6.6 (28–89)	−7.8	2.918	0.004
LDL-C (mg/dl) (min–max)	128.4 ± 38.4 (12–221.6)	103.2 ± 34.5 (28.6–214.4)	21.8	−3.754	<0.001

*All values are expressed as mean ± SD*.

*HDL-C, high-density lipoprotein cholesterol; LDL-C, low-density lipoprotein cholesterol*.

### Urine Albumin, Creatinine, ACR, and eGFR in Patients with and without DR

Urinary albumin concentration (*p* = 0.034) and ACR (*p* = 0.026) were significantly higher and eGFR (*p* = 0.003) was significantly lower in patients with DR compared with patients without DR (Table 4).

**Table 4:** Urinary albumin, creatinine, and eGFR of the study population.

Variable	T2DM		% difference	*t*-Test	*p*-Value
	Retinopathy (*n* = 37)	No retinopathy (*n* = 113)			
Urinary albumin (mg/dl) (min–max)	155.9 ± 217.1 (19.5–1,063)	88.5 ± 145.4 (3–740)	55.2	−2.146	0.034
Urinary creatinine (mg/dl) (min–max)	101.3 ± 30.8 (63–233)	104.1 ± 48.1 (22–347)	−2.7	0.333	0.739
ACR (mg/g) (min–max)	167.4 ± 259.2 (2.8–1,328)	91.9 ± 140.3 (1.9–670)	58.2	−2.254	0.026
eGFR (ml/min/1.73 m^2^) (min–max)	137.8 ± 28.1 (88.5–195.8)	172.3 ± 67.5 (60.9–473)	−22.3	3.013	0.003

*All values are expressed as mean ± SD*.

*ACR, albumin creatinine ratio; GFR, glomerular filtration rate*.

### Logistic Regression Model for Independent Variables Predicting DR

The factors predicting DR were duration of diabetes [OR = 1.143, 95% CI (1.07–1.222), *p* = 0.001], smoking [OR = 3.213, 95% CI (1.395–7.396), *p* = 0.006], family history [OR = 2.559, 95% CI (1.108–5.913), *p* = 0.028], CVD [OR = 4.177, 95% CI (1.398–12.479), *p* = 0.01], nephropathy [OR = 2.225, 95% CI (1.038–4.766), *p* = 0.040], neuropathy [OR = 4.177, 95% CI (1.398–12.479), *p* = 0.01], recurrent infections [OR = 3.452, 95% CI (1.039–11.461), *p* = 0.043], glucose [OR = 1.006, 95% CI (1.002–1.01), *p* = 0.008], urea [OR = 1.102, 95% CI (1.043–1.163), *p* < 0.001], creatinine [OR = 18.028, 95% CI (2.096–155.035), *p* = 0.008], cholesterol [OR = 1.016, 95% CI (1.005–1.026), *p* = 0.004], HDL-C [OR = 0.878, 95% CI (0.807–0.956), *p* = 0.003], LDL-C [OR = 1.02, 95% CI (1.008–1.032), *p* = 0.001], urinary albumin [OR = 1.002, 95% CI (1.00–1.004), *p* = 0.044], ACR [OR = 1.002, 95% CI (1.00–1.004), *p* = 0.041], and eGFR [OR = 0.986, 95% CI (0.977–0.996), *p* = 0.005] (Table 5). The multivariate logistic regression analysis model demonstrated an association between DR with duration of diabetes [OR = 1.114, 95% CI (1.036–1.199), *p* = 0.004], family history of diabetes [OR = 3.397, 95% CI (1.245–9.27), *p* = 0.017], recurrent infections [OR = 0.161, 95% CI (0.033–0.796), *p* = 0.025], and urea [OR = 1.09, 95% CI (1.027–1.157), *p* = 0.005] (Table 6).

**Table 5:** Univariate logistic regression analysis model for independent factors associated with diabetic retinopathy.

Factor	*B*	SE	Wald	*p*-Value	Odds^a^	95% CI for OR	
						Lower	Upper
Duration (years)	0.134	0.034	15.528	<0.001	1.143	1.070	1.222
Smoking	1.167	0.425	7.526	0.006	3.213	1.395	7.396
Family history	0.940	0.427	4.837	0.028	2.559	1.108	5.913
CVD	1.430	0.558	6.556	0.010	4.177	1.398	12.479
Nephropathy	0.800	0.389	4.231	0.040	2.225	1.038	4.766
Neuropathy	1.430	0.558	6.556	0.010	4.177	1.398	12.479
Recurrent infections	1.239	0.612	4.093	0.043	3.452	1.039	11.461
Glucose (mg/dl)	0.006	0.002	7.055	0.008	1.006	1.002	1.010
Urea (mg/dl)	0.097	0.028	12.192	0.000	1.102	1.043	1.163
Creatinine (mg/dl)	2.892	1.098	6.939	0.008	18.028	2.096	155.035
Cholesterol (mg/dl)	0.016	0.005	8.454	0.004	1.016	1.005	1.026
HDL-C (mg/dl)	−0.130	0.043	9.054	0.003	0.878	0.807	0.956
LDL-C (mg/dl)	0.020	0.006	11.659	0.001	1.020	1.008	1.032
Urinary albumin (mg/dl)	0.002	0.001	4.044	0.044	1.002	1.000	1.004
ACR (g/mg)	0.002	0.001	4.183	0.041	1.002	1.000	1.004
eGFR (ml/min/1.73 m^2^)	−0.014	0.005	7.972	0.005	0.986	0.977	0.996

*CVD, cardiovascular disease; HDL-C, high-density lipoprotein cholesterol; LDL-C, low-density lipoprotein cholesterol; GFR, glomerular filtration rate*.

*^a^Adjusted odd ratio*.

**Table 6:** Multivariate logistic regression analysis model for independent factors associated with diabetic retinopathy.

Factor	*B*	SE	Wald	*p*-Value	Odds^a^	95% CI for OR	
						Lower	Upper
Duration (years)	0.108	0.037	8.496	0.004	1.114	1.036	1.199
Family history	1.223	0.512	5.704	0.017	3.397	1.245	9.270
Recurrent infections	−1.827	0.816	5.018	0.025	0.161	0.033	0.796
Urea (mg/dl)	0.086	0.030	7.961	0.005	1.090	1.027	1.157
Constant	−3.383	1.107	9.346	0.002	0.340		

*^a^Adjusted odd ratio*.

### Multiple Linear Regression Model to Predict the Duration of Diabetes

Multiple linear regression analysis demonstrated that duration of diabetes was related to glucose (*t* = 6.646, *p* < 0.001), urea (*t* = 4.355, *p* < 0.001), creatinine (*t* = 3.474, *p* = 0.001), ACR (*t* = 1.981, *p* = 0.049), and eGFR (*t* = −3.519, *p* = 0.001) (Table 7).

**Table 7:** Multiple linear regression model to predict duration of diabetes.

Predictors	*B*	SE	*t*	*p*-Value	95% CI for *B*	
					Lower	Upper
Glucose (mg/dl)	0.033	0.005	6.646	<0.001	0.023	0.043
Urea (mg/dl)	0.236	0.054	4.355	<0.001	0.129	0.343
GFR (ml/min/1.73 m^2^)	−0.026	0.007	−3.519	0.001	−0.040	−0.011
Creatinine (mg/dl)	8.433	2.428	3.474	0.001	3.636	13.231
ACR (g/mg)	0.005	0.003	1.981	0.049	0.000	0.010

*LDL-C, low-density lipoprotein cholesterol; GFR, glomerular filtration rate; T2DM, type 2 diabetes mellitus; B, regression coefficient*.

## Discussion

To date, there has only been one published study on retinopathy in patients with type 2 diabetes in the Gaza Strip (18). The present study is the first to assess the prevalence of DR in men with T2DM in the Gaza Strip in relation to clinical and metabolic factors as well as other complications.

The prevalence of DR in our sample of 150 clinic patients with T2DM was 24.7%, which is comparable with the prevalence of DR in a large population-based retinal screening study in Wales (32). Clearly, further research is required on a larger sample size at a national level. DR was associated with smoking and a family history of diabetes and smoking and family history of diabetes are associated with the development and progression of DR (33).

Diabetic retinopathy was associated with the duration of diabetes (34, 35) and other microvascular and macrovascular complications (36–38). DR as expected was related to poorer glycemic control and lipid profile as well as worsening renal function (38–40). Dyslipidemia has been reported to be associated with CVD and DR (41) and indeed CVD was also related to DR.

The presence of DR was related to increased urinary albumin concentration and ACR as well as a reduced eGFR (18, 42) confirms the association between microvascular complications. Indeed, increased ACR and low eGFR are independently associated with DR. This is important as these tests are relatively cheaper and therefore more affordable compared with ophthalmic assessment, which is not performed routinely in the majority of people in poor areas such as the Gaza Strip. Univariate logistic regression analysis revealed that DR was predicted by duration of diabetes, smoking, family history, CVD, nephropathy, neuropathy, recurrent infections, glucose, urea, creatinine, cholesterol, LDL-C, HDL-C, ACR, and eGFR. Most of these factors have previously been identified as predictive factors in the progression of DR (43). The multivariate logistic regression model revealed that DR was predicted by the duration of diabetes, family history, recurrent infections, and urea. Multiple linear regression also demonstrated that the duration of diabetes was related to glucose, urea, eGFR, creatinine, and ACR.

Limitations of the current study include a small sample size of male patients with the diagnosis of DR being limited to direct ophthalmoscopy. However, this study provides the first set of data on the prevalence of DR among diabetic patients in the Gaza Strip. It also confirms the association of DR with a number of modifiable risk factors including glycemic control, lipids, and impaired renal function as well as a relationship to the other microvascular complications of diabetic neuropathy and nephropathy.

## Ethics Statement

The study was conducted in accordance with the Declaration of Helsinki and was approved by the Local Ethics Research Committee. All subjects provided written informed consent prior to the study.

## Author Contributions

AM data collection, statistical analysis, and write manuscript.

## Conflict of Interest Statement

The author declares that the research was conducted in the absence of any commercial or financial relationships that could be construed as a potential conflict of interest. The reviewer GP and handling editor declared their shared affiliation.
